# Effects of prolactin on movement disorders and APOE, GFAP, and PRL receptor gene expression following intracerebral hemorrhage in rats

**DOI:** 10.22038/IJBMS.2021.58176.12927

**Published:** 2021-12

**Authors:** Shiba Yousefvand, Mousa-al-Reza Hadjzadeh, Zakieh Keshavarzi, Hamid Dolatshad, Farzaneh Vafaee, Maryam Mahmoudabady, Zahra Gholamzadeh Virany

**Affiliations:** 1 Department of Physiology, Faculty of Medicine, Mashhad University of Medical Sciences, Mashhad, Iran; 2 Division of Neuro-Cognitive Sciences, Psychiatry and Behavioural Sciences Research center, Mashhad University of Medical Sciences, Mashhad, Iran; 3 Neurogenic Inflammation Research Center, Mashhad University of Medical Sciences, Mashhad, Iran; 4 Department of Physiology, School of Medicine, North Khorasan University of Medical Sciences, Bojnurd, Iran; 5 Division of Clinical Laboratory Science, Radcliffe Department of Medicine, University of Oxford, Oxford, UK; 6 Neuroscience Research Center, Mashhad University of Medical Sciences, Mashhad, Iran; 7 Department of Neuroscience, Faculty of Medicine, Mashhad University of Medical Sciences, Mashhad, Iran; 8 Department of Biology, Faculty of Sciences, Islamic Azad University-Mashhad Branch, Mashhad, Iran

**Keywords:** ApoE, GFAP, ICH, PRL receptor, Striatum

## Abstract

**Objective(s):**

Intracerebral hemorrhage (ICH) occurs mostly in the striatum. In ICH, blood prolactin level increases 3-fold. The effects of intracerebroventricular injection (ICV) of prolactin on motor disorders will be investigated.

**Materials and Methods:**

This study was performed on 32 male Wistar rats in 4 groups: sham, ICH, and prolactin with 1 μg/2 μl (P1) and 2 μg/2 μl (P2) doses.

**Results:**

The weight of animals on days 1 (*P*˂0.01), 3, and 7 (*P*˂0.05) in the sham and P2 groups increased compared with the ICH group. Neurological Deficit Score (NDS) in ICH and P1 groups decreased, and increased compared with sham and ICH groups (*P*˂0.001), respectively. NDS in the P1 group increased compared with the P2 group on days 1 (*P*˂0.0 5), 3, and 7 (*P*˂0.001). The duration time of rotarod in ICH and P1 groups decreased and increased compared with sham and ICH groups (*P*˂0.001), respectively. The duration time of rotarod in the P1 group on days 3 and 7 increased compared with the P2 group (*P*˂0.001). Travel distance in days 1(*P*˂0.01), 3(*P*˂0.001), and 7(*P*˂0.01) decreased in the ICH group. Prolactin receptor (PRL receptor) expression in ICH, P1, and P2 groups increased compared with sham and ICH groups (*P*˂0.001). Glial fibrillary acidic protein (GFAP) expression (*P*˂0.001) and apolipoprotein E (APOE) (*P*˂0.01) expression in the ICH group increased compared with the sham group. GFAP and APOE expression in the P1 group increased compared with the ICH group (*P*˂0.001). APOE expression in the P1 group increased compared with the P2 group (*P*˂0.001).

**Conclusion:**

According to the results, prolactin reduces movement disorders.

## Introduction

Stroke occurs due to a brain region’s lack of blood flow and the consequent loss of part of the neuronal activities. Stroke is the third leading cause of death worldwide. More than five and a half million people die due to stroke worldwide per year (1). Also, stroke leads to motor and cognitive impairments; of which, of course, motor impairments are more noticeable than cognitive impairments, and more focus is on correcting movement disorders (2). Types of stroke include transient ischemic attack, ischemic stroke, and intracerebral hemorrhage (ICH) (1). ICH, which is caused by cerebral artery bleeding, is less frequent but is very dangerous (3, 4). Several factors are involved in the occurrence of ICH, but the most important is high blood pressure (1). ICH most often occurs in the striatum (5). The striatum receives input from all regions of the cerebral cortex, thalamus, and limbic system (6). The striatum has two-way communication with the cortex. As a result of these connections, damage to the striatum leads to impaired motor and cognitive functions, as well as damage to other regions of the brain such as the cortex and limbic system (7). After a stroke, the levels of blood factors such as prolactin, cortisol, T3 level**,** and some others increase. Among these factors, blood prolactin levels increase threefold in comparison with normal status (8-10).

Prolactin was isolated from the anterior pituitary gland in 1931 and purified. Prolactin has several functions in the central nervous system (CNS) (11, 12). Prolactin is present in various regions of the brain via transporter proteins. Prolactin enters the brain through the circulation with the carrier to carry out most of its functions in the CNS (13). Prolactin is known as an anti-stress hormone and its secretion increases in response to widespread stressors. Acute brain damage has been shown to up-regulate prolactin mRNA expression in the brain (14-16). When damage occurs in the cerebral cortex, the levels of prolactin and its receptors in the neurons and astrocytes in the affected region rise. Prolactin exerts its protective effects on brain damage, possibly by acting on glial cells (astrocytes) (17, 18). In ischemia, prolactin increases the expression of the glial fibrillary acidic protein (GAFP) and increases the viability of astrocytes by acting on astrocytes. GAFP is a protein and serves in increasing the activities of astrocytes (19). Apolipoprotein E (APOE) expression also increases in astrocytes after acute brain injury. This protein promotes neuronal growth/synaptogenesis, amyloid clearance, regulation of the neuro-immune response, and cerebrovascular function. APOE receptors are located on the nerve cells (20). APOE is primarily produced and secreted by astrocytes and affects immune responses including cell-cell communication and migration inside and outside the CNS (21).

Due to the effects of stroke on movement disorders and the consequent effect on the person’s social functioning, several treatments have been investigated to treat ICH, but these treatments have a long treatment period, have many side effects, and in many cases do not completely cure the person’s mobility defects. Prolactin has been shown to have protective effects on brain damage, possibly through astrocytes. No side effects have been reported with the use of prolactin in the treatment of brain damages. To date, no study has been performed on the therapeutic effects of prolactin in the improvement of movement disorders caused by ICH. Therefore, in this study, the protective effects of prolactin in the treatment of motor injuries caused by ICH and its effect on the expression of genes related to astrocyte activities, including GFAP and APOE, are investigated. Also, in this study, the effect of exogenous prolactin on the expression of prolactin receptors (PRL receptor) in the striatum will be investigated.

## Materials and Methods

***Animals***

This study was conducted on 32 adult male Wister rats weighing 200–250 g. The animals were obtained from the Animal Home Center at Mashhad University of Medical Sciences, Iran. These animals were kept in standard conditions (22 ± 2 °C, 12 hr of darkness/12 hr of light, and 40% humidity), and during the study had free access to food and fresh water. The experiments were performed according to guidelines proposed by the National Institutes of Health Guide for the Institutional Animal Care (22), and the Institutional Ethics Committee at Mashhad University of Medical Sciences (ethical code: IR.MUMS. MEDICAL. REC.1399.026). Five days before the experiments, the animals were placed in the laboratory for half an hour and handled. On day 6, intracerebral hemorrhagic model surgery was performed on animals. In this study, 8 animals were used in each group, but finally, the results of 6 animals (as a result of correct injections) were included in the statistical analysis.

***Surgical producer***

Animals were anesthetized by intraperitoneal injection of xylazine (10 mg/kg, IP) and ketamine (100 mg/kg, IP), and then placed in a stereotaxic apparatus. After opening the scalp and exposing the skull, the exact location of the striatum using the Paxinus atlas was determined as follows: 0.36 mm anterior (AP), 3.4 mm lateral (ML), and 6 mm ventral (DV) coordinates (23). Then 100 microliters of fresh animal blood were taken from the sinus behind the eye (24-26), and this fresh blood was injected unilaterally into the left striatum using a Hamilton needle syringe (100 μl, 700 sequences, Hamilton Corporation, Switzerland) (27). Then, half an hour after the injection of fresh blood into the striatum, the coordinates of the right ventricle were determined according to the Paxinus atlas as follows: 0.72 mm anterior (AP), 1.4 mm lateral (ML), and 4.8 mm ventral (DV) coordinates (23, 27). After that, prolactin was injected into the right ventricle (28). Finally, the scalp was sutured with 03 silk sutures and the animals were cared for under standard animal conditions.

***Experimental procedure***

32 male rats were randomly divided into 4 groups (8 per group). Sham group: in this group, all conditions were similar to intervention groups, except for intervention factors (ICH induction and prolactin injection). For this purpose, rats in this group underwent only surgery without ICH induction and received a vehicle injection (artificial CSF) into their ventricles after 30 min. Behavioral tests were taken on these rats on days 1, 3, and 7 after surgery, and the weights of the animals on days 1, 3, and 7 after surgery were measured. ICH group: this group was the control group. In this group, ICH intervention was performed, but the animals were not treated. For ICH induction, 100 microliters of fresh blood were injected into the striatum with a Hamilton needle syringe. 30 min after surgery, a vehicle was injected into the ventricle. Behavioral tests were performed, and the weight of the animals was also measured as in the previous group. Prolactin group (1 μg/2 μl ): in this group, to evaluate the effect of prolactin on ICH, a dose of 1 μg/2 μl of prolactin was used as treatment (29, 30). Rats in this group underwent ICH induction surgery; 30 min after surgery, a dose of 1 μg/2 μl of prolactin was injected into the ventricle. Behavioral tests were performed, and the weight of the animals was also measured as in the previous group. Prolactin group (2 μg/2 μl ): this group was treated similar to the Prolactin group (1 μg/2 μl ), but only the dosage of prolactin was 2 μg/2 μl (29, 30).

***Behavioral tests***

*Neurological deficit score*

All six factors of neurological deficit score (NDS) including body symmetry, climbing, gait, circling behavior, compulsory circling, and front limb symmetry were calculated after 1, 3, and 7 days after surgery. Each of these factors was assigned a score of 3 (lowest) to 18 (highest) (27).

*Rotarod test*

The rotarod device is subjected to an accelerator protocol of 4-40 rpm, and it is used to evaluate motor and balance behavior. In this test, the animals need to be trained before surgery. For this purpose, animal training was started 4 days before surgery. For 3 days, the animals were placed inside the device by the tester at 10 rpm for 5 min. On the fourth day, the animals were tested. In this way, the animals were tested 3 times for 5 min and the animal was given a half-hour rest between each test, then the average was reported (31). Finally, the animals were re-tested on days 1, 3, and 7 after surgery.

***Open field test***

After ICH-induction, the open field test (OF) is used to check the movement defects. This test consists of a box with dimensions (60x45x45 cm) that is connected to a special software by the camera. The bottom of the box is divided into different parts by electric fuses. On days 1, 3, and 7 after surgery, the animal is placed inside the box and the animal’s passage in this box is recorded by the software for 15 min (32).

*Gene expression*

*Tissue collection for conducting Real-Time PCR*

After deep anesthesia, the animals were decapitated, and the brain was separated immediately. Then, their left striatum was isolated and immediately frozen with liquid nitrogen inside microtubes and stored at -80 °C for gene expression assessment.

*RNA extraction*

To the tissues inside the microtubes, the first 1 µl ice-cold of RNX plus (Sinaclon, Iran) solution was added and homogenized well. After 5–10 sec vortexing, the homogenates were incubated at room temperature for 5 min. Then, 200 µl chloroform (Hamoon Teb Co, Iran) was added to the vial. After shaking the mixture for 15 sec (no vortexing) the vial was incubated in ice for 5 min. The mixture was centrifuged for 15 min at 12,000×g at 4 °C (MPW-35IR, Portland). In this stage, three phases were created. Carefully the upper phase (RNA phase) was removed and transferred to the RNaes-free microtube. Isopropanol (Hamoon Teb Co, Iran) was added to the vial in an equal volume of the water phase volume. The vial was shaken vigorously to mix the contents, and then incubated on ice for 15 min and centrifuged for 15 min at 12,000×g at 4 °C. The supernatant was discarded and the precipitate dissolved, which contained RNA, in 1 ml of 75% ethanol (Hamoon Teb Co, Iran). Then the vial was centrifuged for 8 min at 7500×g at 4 °C. The supernatant was discarded and the precipitate was incubated at room temperature for a few min until the alcohol evaporated and dried. Depending on the amount of sediment and extracted RNA, the sediment was dissolved in 20–50 µl DEPC water (Sinagen, Iran), and heated in a 55–60 °C water bath (Behdad, Iran) for 10 min and ultimately they were stored at -80 °C until use. The nanodrop spectrophotometer (Thermo Scientific, USA) (260λ, ultraviolet light) was used to measure the quantity of RNA, 1 μl of RNA was diluted in 50 µl of DEPC-treated water. The stock concentration was obtained based on 1OD = 40 μg/ml of RNA and dilution of 1/100.

*cDNA synthesis*

Easy ^TM^ cDNA Synthesis Kit (Parstous, Inc., Iran, Cat NO. A101161) was utilized for cDNA synthesis using C1000^TM^ Thermal Cycler (BIO-RAD, USA). Template RNA and other kit components were mixed in RNase-free microtubes as explained: Template RNA+ Buffer-Mix (2x) (10 µl) +Enzyme-Mix (2 µl) +DEPC-treated water (Up to 20 µl). Incubation was done for 10 min at 25 °C and 60 min at 45 °C. The reaction was stopped by heating the mixture for 5 min at 85 °C and finally, it was chilled on ice or at 4 °C. 

*Quantitative real-time polymerase chain reaction (QRT-PCR)*

The StepOnePlus™ thermocycler (Applied Biosystems, USA) was used to process the reactions in a 48-well plate for real-time PCR interaction. Each microtube in each plate with 48 wells including SYBR™ green (10 µl) of PCR master mix, cDNA (2 µl), the rest of nuclease-free water (7 µl), and finally 0.5 µl forward and 0.5 µl reverse primers. PCR primers were designed using Gene Runner (Version 6.5.51, free online Software, Inc., Hudson, USA) online software and controlled by the NCBI and Gene runner to ensure no cross-reactivity. The housekeeping β-actin gene was run independently under the same experimental conditions in both reactions to obtain the integrity and quantity of RNA at the start of the real-time reaction. The rat β-actin (forward sequence of 5′-AACCCTAAGGCCAACCGTG - 3′ and reverse sequence of 5′-TACGTACATGGCTGGGGTGT -3, rat GFAP (forward sequence of 5′-CGAGTCCTTGGAGAGGCAAA - 3′ and reverse sequence of 5′-TACAGGAATGGTGATGCGGT - 3′, rat APOE (forward sequence of 5′-CCAGGGGGCTTGACTGG - 3′ and reverse sequence of 5′-TTCCTGTGTGACTTGGGAGC - 3′, and rat PRL receptor (forward sequence of 5′-TACATCGTTGAGCCAGAGCC - 3′ and reverse sequence of 5′-TCAGTTATGGTGGGTGGGGA - 3′, were the primers used in the PCR process. The protocol of the PCR procedure was as the following order: denaturation at 95 °C for 15 min, 40-cycle amplification with denaturation (95 °C, 35 sec), annealing (60 °C, 35 sec), and extension (72 °C, 35 sec). A melting curve analysis determined the specificity of PCR products. The experimental threshold cycle (Ct) was calculated by the algorithm improvements supplied by the equipment. The samples were checked twice and an interpretation of the measured mean values was carried out. Using default parameters, the Ct values were obtained in each reaction with the help of instruments. Relative gene expression (fold changes) was calculated using the 2^−ΔΔCT^ method (33). To monitor APOE, GFAP, PRL receptor, and β-actin levels, the reactions proceeded independently in the tubes with the same samples. 

***Statistical analysis***

All data in this study were evaluated using the SPSS 16.0 (SPSS Inc., IBM) software package. The data were presented as Mean±SEM. For evaluating the results, repeated measures ANOVA was used, and for confirmation of intergroup comparison, one-way ANOVA with *post hoc* Tukey’s test were used. The non-parametric Mann-Whitney test was applied to statistically analyze NDS. The statistical significance level was *P*<0.05. 

## Results

**Figure 1:**
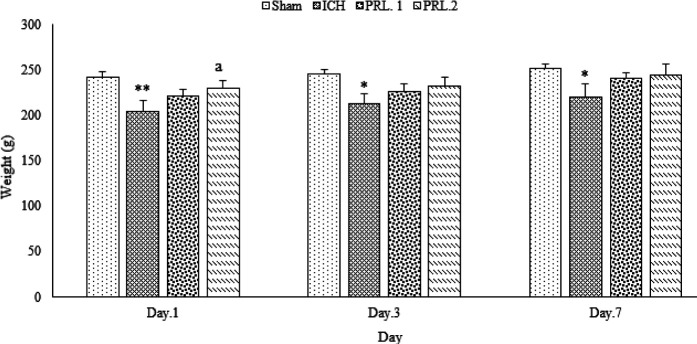
Effects of ICV injection of prolactin (1 μg/2 μl and 2 μg/2 μl) on the weight of rats with ICH. Data are expressed as Mean±SEM. * P˂0.05, ** *P*˂0.01 (ICH compared with Sham group). a, * P*˂0.05 (ICH compared with the treated groups) (N= 6 per group)

**Figure 2:**
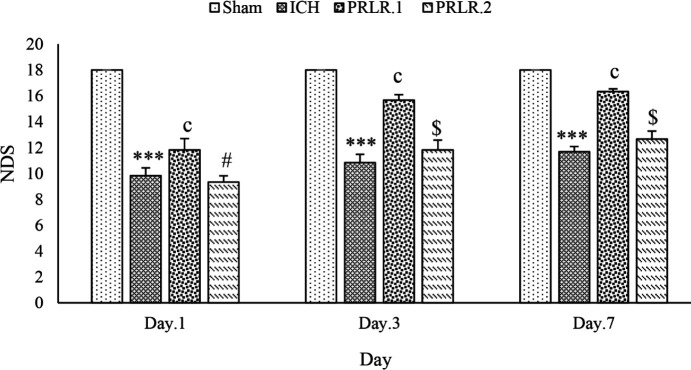
Effects of ICV injection of prolactin (1 μg/2 μl and 2 μg/2 μl ) on the NDS of rats with ICH. Data are expressed as Mean±SEM. *** *P*˂0.001 (ICH compared with Sham group). c, *P*˂0.001 (ICH compared with the treated groups). # *P*˂0.05, $ *P*˂0.001 (Treatment groups compared with each other) (N= 6 per group)

**Figure 3:**
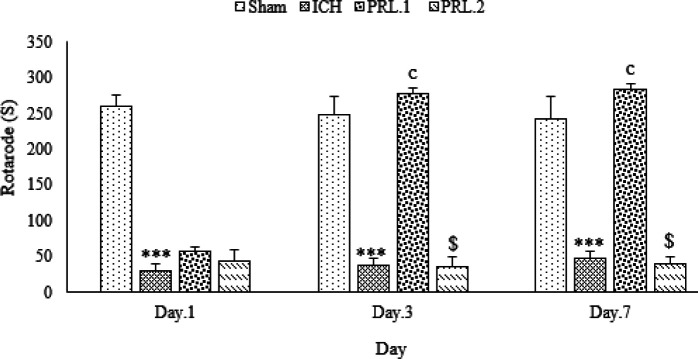
Effects of ICV injection of prolactin (1 μg/2 μl and 2 μg/2 μl ) on the rotarod test in rats with ICH. Data are expressed as Mean±SEM. *** P˂0.001 (ICH compared with Sham group). c, * P*˂0.001 (ICH compared with the treated groups). $ * P*˂0.001 (Treatment groups compared with each other) (N= 6 per group)

**Figure 4:**
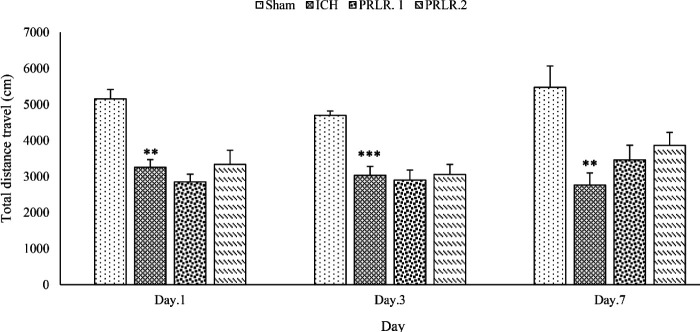
effects of ICV injection of prolactin (1 μg/2 μl and 2 μg/2 μl) on the travel distance in rats with ICH. Data are expressed as Mean±SEM. *** *P*˂0.01 and *** *P*˂0.001 (ICH compared with the Sham group). (N= 6 per group)

**Figure 5:**
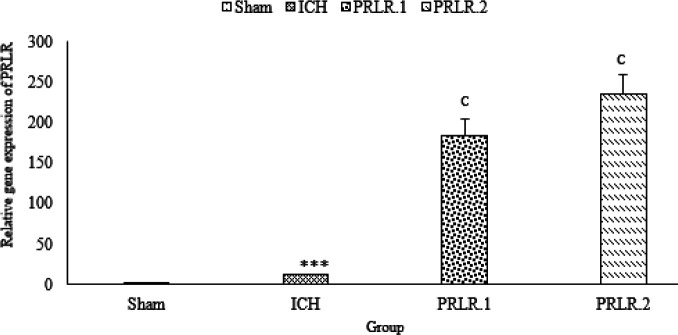
Effects of ICV injection of prolactin (1 μg/2 μl and 2 μg/2 μl ) on prolactin receptor (PRL receptor) expression of rats with ICH. Data are expressed as Mean±SEM. *** *P*˂0.001 (ICH compared with Sham group). c, *P*˂0.001 (ICH compared with the treated groups) (N= 6 per group)

**Figure 6:**
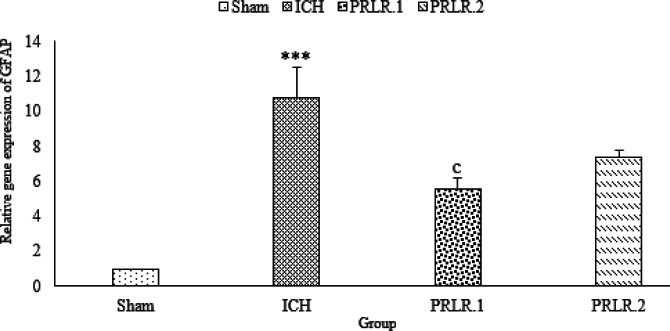
Effects of ICV injection of prolactin (1 μg/2 μl and 2 μg/2 μl ) on the Glial fibrillary acidic protein (GFAP) expression of rats with ICH. Data are expressed as Mean±SEM. *** *P*˂0.001 (ICH compared with Sham group). c, *P*˂0.001 (ICH compared with the treated groups). (N= 6 per group)

The results of the effects of intracerebroventricular injection (ICV) injection of prolactin on the weight of animals, movement disorders, degree of neurological defects, as well as PRL receptor, GFAP, and APOE gene expression on days 1, 3, and 7 after surgery in rats were shown in Figures 1–6.

*Behavioral test assessment.*

The weight of animals on days 1 (*P*˂0.01), 3, and 7 (for both, *P*˂0.05) after surgery was significantly reduced in the ICH group compared with the sham group. Also, the weight of animals in the prolactin-treated group with THE 2 μg/2 μl dose on day 1 after surgery, increased significantly compared with the ICH group (*P*˂0.05) (Figure 1).

On days 1, 3, and 7 after surgery, NDS in the ICH group significantly decreased in comparison with the sham group (for all groups, *P*˂0.001). NDS in the treated groups with prolactin at a dose of 1 μg/2 μl on days 1, 3, and 7 significantly increased compared with the ICH group (for all groups, *P*˂0.001), but NDS in the prolactin treated group with 2 μg/2 μl dose in days 1, 3 and 7 was not significantly different compared with the ICH group (*P*>0.05). The NDS score in the prolactin treated group with 1 μg/2 μl dose was significantly increased compared with the prolactin treated group with 2 μg/2 μl dose on days 1 (*P*˂0.05), 3, and 7 (for both, *P*˂0.001) after surgery (Figure 2).

The duration time of the stay of animals on the rotarod in the ICH group on days 1, 3, and 7 after surgery was significantly reduced compared with the sham group (for all groups, *P*˂0.001). The duration time of stay of animals on the rotarod in the prolactin treated group with 2 μg/2 μl dose was not significantly different from the ICH group on days 1, 3, and 7 (*P*>0.05), also in the prolactin treated group with 1 μg/2 μl dose, the duration time of stay on the rotarod was not significantly different from that of the ICH group (*P*>0.05). The duration time of stay on the rotarod on days 3 and 7 after treatment in the prolactin treated group with 1 μg/2 μl dose was significantly increased compared with the ICH group (for both, *P*˂0.001). The duration time of stay on rotarod in prolactin treated group with 1 μg/2 μl dose on days 3 and 7 after treatment was significantly increased compared with prolactin treated group with 2 μg/2 μl dose (for both, *P*˂0.001) (Figure 3).

The travel distance in OF box in the ICH group on days 1 (*P*˂0.01), 3 (*P*˂0.001), and 7 (*P*˂0.01) after surgery was significantly decreased compared with the sham group. The travel distance in OF box in both prolactin-treated groups was not significantly different from the ICH group on days 1, 3, and 7 (*P*>0.05) (Figure 4).

*Gene expression*

As shown in Figure 5, PRL receptor mRNA expression in the ICH group significantly increased compared with the sham group (*P*˂0.001), PRL receptor gene expression in both of prolactin treated groups (1 μg/2 μl and 2 μg/2 μl ) had a significant increase compared with the ICH group (for both, *P*˂0.001) (Figure 5).

In Figure 6, GFAP gene expression in the ICH group had a significant increase compared with the sham group (*P*˂0.001), also, GFAP gene expression in the prolactin treated group with 1 μg/2 μl dose significantly increased compared with the ICH group (*P*˂0.001), but GFAP gene expression in the prolactin treated group with 2 μg/2 μl dose did not change significantly compared with the ICH group (*P*>0.05) (Figure 6).

**Figure 7:**
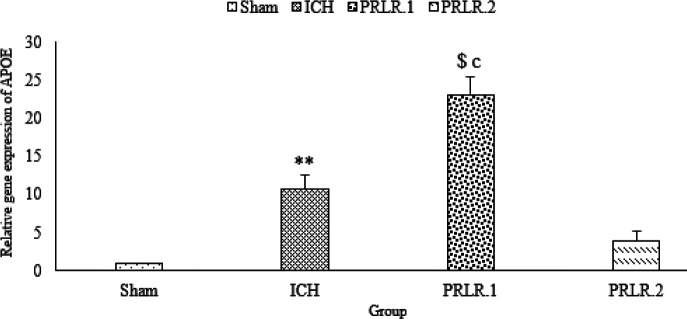
Effects of ICV injection of prolactin (1 μg/2 μl and 2 μg/2 μl ) on Apolipoprotein E (APOE) expression in rats with ICH. Data are expressed as Mean±SEM. ** *P*˂0.01 (ICH compared with Sham group). c, *P*˂0.001 (ICH compared with the treated groups). $ *P*˂0.001 (treatment groups compared with each other) (N= 6 per group)

Figure 7 indicated that APOE gene expression in the ICH group significantly increased in comparison with the sham group (*P*˂0.01). Also, APOE gene expression in the prolactin group with 1 μg/2 μl dose significantly increased compared with the ICH group (*P*˂0.001). APOE gene expression in prolactin group with 1 μg/2 μl dose significantly increased compared with the prolactin group with 2 μg/2 μl dose (*P*˂0.001) (Figure 7).

ICV: Intracerebroventricular; ICH: Intracerebral hemorrhage

ICV: Intracerebroventricular; ICH: Intracerebral hemorrhage

ICV: Intracerebroventricular; ICH: Intracerebral hemorrhage

ICV: Intracerebroventricular; ICH: Intracerebral hemorrhage

ICV: Intracerebroventricular; ICH: Intracerebral hemorrhage

ICV: Intracerebroventricular; ICH: Intracerebral hemorrhage

ICV: Intracerebroventricular; ICH: Intracerebral hemorrhage

## Discussion

The results of this study showed that ICV injection of prolactin 30 min after ICH improved movement disorders in rats. Also, among the therapeutic doses used (1 μg/2 μl and 2 μg/2 μl), the dose of 1 μg/2 μl had the greatest effect on the improvement of movement disorders. The results of the PRL receptor, GFAP, and APOE gene expression also confirmed the results of improving movement disorders in the behavioral section of the study. The golden time in individuals with ICH is between 30 min to 4 hr after ICH, and with a proper performance at this time, the situation can be improved in these individuals. In the absence of proper performance at this time, the induced disorders persist throughout life and adversely affect a person’s personal and social life (2, 34). Therefore, in this study, for optimal function, prolactin was injected into the right ventricle 30 min after ICH. Also, considering that most often ICH occurs in the striatum, and of course due to the two-way communication of the striatum with the cortex, and the role of the striatum in the processing of motor functions (6, 7), in this study ICH was inducted in the striatum. 

The weight of rats in the ICH group decreased, but in the treated group (2 μg/2 μl ) increased on the first day after ICH. Consistent with the results of the present study, it was reported that people with traumatic brain injury (TBI) lose weight due to hormonal disorders that occur, such as adrenal insufficiency, or disorders in the neural networks associated with food intake in the brain (35, 36). In the prolactin-treated group, this weight loss was compensated for on the first day after treatment and may indicate the protective effects of prolactin on post-ICH disorders. During brain injury, food intake is reduced due to neurological defects and damage to the neural networks involved in central controlling food intake. By reducing the animal’s food intake, the animal’s weight also decreases (37). According to the results of this study, on day 1 after ICH, the rate of neurological defects decreased by ICV injection of prolactin and subsequently increased the NDS score. This increase in the NDS score means improvement of neurological defects. Therefore, following the improvement of neurological defects, the animal’s food intake may have increased compared with the ICH group. This increase in food intake may lead to an increase in the animal’s weight on day 1 after in the prolactin group. The most increase in the NDS score and the therapeutic effects on the improvement of movement disorders were observed in the treated group with 1 μg/2 μl dose of prolactin. These results indicate the effectiveness of this dose on improvement of neurological defects in animals with the experimental model of ICH. Also, these results in the behavioral section are associated with the results of gene expression in the molecular section. In the behavioral section, the most therapeutic effects, and also the highest level of expression in the gene expression section were observed with1 μg/2 μl dose of prolactin. These results indicate that in this study, 1 μg/2 μl dose of prolactin is the optimal effective dose.

 Consistent with the results of the present study, it has been shown that when brain damage occurs, PRL receptor levels in the astrocytes in the affected regions rise, and prolactin probably exerts its protective effects through glial cells (17, 18). It has also been shown that prolactin in the cell culture medium, increases GFAP gene expression and increases PRL receptor expression because by removing prolactin from the culture medium, the PRL receptor and GFAP gene expression were reduced (38). During brain injury, PRL receptor expression in astrocytes in the affected region increases (39), and thus it seems that prolactin exerts its protective effect in this way (40). Therefore, it can be relevant to conclude that, ICV injection of prolactin after ICH resulted in increased PRL receptors in affected regions.

In brain damage, prolactin exerts its protective effects through astrocytes (17). It has been shown that in PRL receptor knock out mice, the GFAP expression was less than in mice with the PRL receptor (40). Prolactin in rats with excitatory toxicity induced by neurodegenerative kainic acid (KA) decreased neurodegenerative damage in male rats by increasing GFAP expression (18, 41). It was also indicated that prolactin in ischemia increases GAFP expression and astrocyte survival by acting on astrocytes (19). According to the results of the above studies, and the results of the present study, ICV injection of prolactin after ICH increased PRL receptor expression in the striatum. On the other hand, GFAP expression is increased in astrocytes of the affected regions. Because the marker of astrocyte activity is the GFAP gene, an increase in GFAP is associated with an increase in astrocyte activity, so by these results, prolactin may have exerted its protective effects on the improvement of movement disorders by acting on astrocytes in the affected regions.

APOE is primarily produced and secreted by astrocytes (21). After acute brain injury, APOE expression in astrocytes increases; which promotes neuronal growth/synaptogenesis, amyloid clearance, and regulation of the neuro-immune response (20). APOE has been shown to reduce neurological defects in stroke rat models by increasing APOE2 receptor cholesterol uptake (42). Knockout APOE mice have also been shown to have a higher rate of post-stroke neurological damage than mice with APOE, as well as more engaged affected regions. In these mice, continuous intracerebral injection of APOE improved brain damage (43). APOE is up-regulated after brain injury (29, 44). According to the results of these studies, APOE expression increase is associated with a decrease in nerve damage. In the present study, ICV injection of prolactin increased APOE expression in the affected regions, and movement disorders improved. Therefore, astrocytes via increasing APOE expression may be involved in reduced movement disorders.

## Conclusion

 According to the results of the present study, in cases with ICH, ICV injection of prolactin increased PRL receptor expression in the affected regions, and increased GFAP and APOE genes expression, and caused improved movement disorders in rats. Thus, ICV injection of prolactin may increase PRL receptor expression in the affected regions, and then due to the astrocytes activated in the damaged regions reduce neurodegeneration, resulting in greatly improved movement disorders. Therefore, prolactin injected into the ventricles of the brain can greatly reduce ICH-induced movement disorders. The authors of this study suggest that the signaling pathways associated with the genes studied in this study be further explored. 

## Authors’ Contributions

SY Performed laboratory work, collection of results, and data processing; MRH Designed the study, critically revised and edited the article, approved the final version to be published, supervised, and helped in funding acquisition; ZK Contributed in writing and funding acquisition; HD Contributed in supply of materials and funding acquisition; FV Helped in data processing and performing experiments; MM Helped in laboratory work and designed part of experiments; ZGV Contributed in part of laboratory work. All authors read and approved the final version of the manuscript.

## Conflicts of Interest

The authors do not have any conflicts of interest.
